# The Relationship between Achievement Motivation and Job Performance among Chinese Physicians: A Conditional Process Analysis

**DOI:** 10.1155/2021/6646980

**Published:** 2021-04-07

**Authors:** Botang Guo, Binbin Qiang, Jiawei Zhou, Xiuxian Yang, Xiaohui Qiu, Zhengxue Qiao, Yanjie Yang, Depin Cao

**Affiliations:** ^1^Department of Medical Psychology, Psychological Science and Health Management Center, Harbin Medical University, Heilongjiang Province, China; ^2^Department of Medical Education, School of Health Management, Harbin Medical University, Heilongjiang Province, China

## Abstract

**Background:**

To explore the relationship between achievement motivation and job performance among physicians, this study investigated the impacts of different personality traits on job performance among the physicians.

**Methods:**

This cross-sectional study was conducted in 2017 and 1,523 physicians from eight tertiary grade A hospitals in Harbin, China. The type of data collected included the achievement motivation of the physicians, job performance, organizational commitment, personality traits, and other demographic variables. To assess and compare the demographic data, independent *t*-test and ANOVA were applied. Further, Pearson correlation coefficients were used to evaluate the correlation among the variables. Moderated mediation analysis was performed to test the correlation among the job performance, achievement motivation, organizational commitment, neuroticism, extraversion, openness, agreeableness, and conscientiousness.

**Results:**

Achievement motivation directly influences job performance and organizational commitment partially mediates the direct effects of achievement motivation on job performance. Additionally, our findings demonstrated that agreeableness and conscientiousness moderate the strength of the relationships between achievement motivation and job performance mediated by organizational commitment.

**Conclusion:**

We propose that hospital managers should pay attention to the personal growth of the physicians and improve their organizational commitment via creating a positive working climate and training for career planning and education. Moreover, managers should identify conscientiousness and agreeableness individuals and increase their responsibilities geared towards improving the performance of the organization.

## 1. Background

With the growing economy, the medical system of China is gradually advancing; yet, there still exist serious challenges. The number of licensed physicians per 1000 people in China was 2.12 in 2014 [1], which was lower than the UK's at 2.8 and the USA's at 2.4 per 1000 and better than India's 0.6 per 1000, respectively [2]. Unlike their counterparts in developed countries, Chinese physicians work under immense pressure. The characteristics and nature of their work are unique since it involves life and health of humans. Notably, Chinese physicians handle multiple tasks, including routine diagnosis, clinical teaching, and scientific research [3]. At the same time, they practice under unfavorable conditions, for instance, frequent medical injury cases, low salaries, and handing voluminous cases of diagnosis and treatment which adversely affect the work attitude of the physicians, causing low work motivation and performance [4, 5]. As a result, this reduction in performance impairs medical services and further triggering dissatisfaction among the patients [6]. Job performance is a parameter that describing all attitudes, behaviors, and task completion level of employees; it is divided into individual performance and organizational performance [7]. Individual performance is often evaluated by superiors and peers [8], while organizational performance refers to the quantity, quality, and efficiency of assigned tasks [9]. Generally, an increase in organizational performance is attributed to high individual performance. Therefore, the job performance of the physicians has undoubtedly become a subject of important focus among hospital administrators and researchers.

Achievement motivation has become one of the essential organizational behaviors that influence job performance. Research evidence shows that within a specific range, work efficiency is enhanced with an increase in motivation intensity [10]. Numerous scholars have confirmed the role of achievement-motivation in job performance [11, 12]. Also, a meta-analysis in the field of public administration showed that intrinsic motivation positively affects job satisfaction and job performance [13]. Notably, unlike in other fields, physicians are trained for a prolonged period in the medical field and they have clear professional goals. They desire to correctly and efficiently complete their tasks in their hospitals. According to the achievement need hypothesis coined by McClelland, people exhibiting high achievement motivation are more devoted to work, and they further achieve higher performance by enjoying an adventurous work environment through positive feedback [14]. Additional research evidence indicates that internal drive and motivation positively influence job performance among Chinese physicians [15]. By the inference, we hypothesized that

*H1*: there is a positive correlation between achievement motivation and job performance among physicians.

Although some studies argue that achievement motivation promotes job performance, the underlying mechanisms of this relationship are controversial. Whether there exists an intermediate variable that plays a crucial role remains unknown.

In recent years, organizational commitment has been an area of interest of enterprises. Organizational commitment is the relative strength of the recognition and participation of individuals in a particular organization [16]. Considerable research evidence shows that employees with high organizational commitment exhibit a strong sense of identity and belonging to their organizations [17, 18]. They take pride in being a member of the particular organization and willing to make sacrifices for the benefit of the organization [19]. Similarly, the theory of social exchange postulates that employees with high organizational commitment have a great sense of participation and dedication, translating to better performance [20]. Elsewhere, findings from a meta-analysis showed that there is a strong association between emotional commitment and job performance [21]. Additional reports suggest that organizational commitment positively influences job performance, embodied in patient care and business effectiveness [22]. Further, a study in England revealed that staff invests more in the hospital, and the financial performance is better [23].

Additionally, employees with high achievement motivation are more connected to the organization [24]. A study in south-west, Nigeria, indicated that achievement motivation and organizational commitment are significantly and positively correlated [25]. Moreover, a cross-sectional study involving university academic staff showed that achievement motivation is positively related to organizational commitment [26]. Achievement motivation constitutes the predecessor of organizational commitment, which in turn is an antecedent variable for significant work outcomes. Therefore, based on this, we speculate that

*H2*: the organizational commitment of doctors mediates the effect of achievement motivation on job performance.

Personality is the internal tendency of individual behaviors. It is manifested in the integration of individual needs, motivation, attitude, personality, and performance when people adapt to environments. A stable personality is formed with the adaptation in a particular environment. Personality psychology postulates that when adapting to organizations and society, people with different personalities exhibit different needs, attitudes, characters, and performances, which affect their learning, life, and work [27, 28]. A literature review on organizational behavior validated the predictive effect of big five personalities on job performance [29]. Among the five types of occupational groups, conscientiousness personality is closely related to the performance in all occupational jobs. Similarly, extroversion, neuroticism, agreeableness, and openness are closely related to the performance in a particular occupation [30]. Additional research evidence confirms that in a task that requires cooperative communication, conscientiousness and agreeableness have a significant impact on job performance [31]. In Indonesia, it was shown that the extrovert personality among physicians results in optimal work performance [32]. Another study showed that personality moderates the coping styles to influence the anxiety symptoms among Chinese physicians [33]. In this study, we speculate that personality plays a similar role. Moreover, information on whether personality moderates the mediating effect of organizational commitment on job performance is unknown. Therefore, the current study used personality as the moderator and hypothesized further that

*H3*: personality moderates the mediating effect of organizational commitment on job performance.

## 2. Methods

### 2.1. Participants and Study Design

This cross-sectional survey was conducted in Harbin City, Heilongjiang Province, located in the northeast of China between March and September 2017. Harbin City has 13 tertiary grade A hospitals, with a bed capacity of over 500 providing both comprehensive and specialized medical care with a high level of medical training and research [34]. Using a random number table, a total of 8 hospitals were selected. Thereafter, the cluster sampling procedure was used to recruit 1,600 physicians from the 8 tertiary grade A hospitals. Data was collected by filling the questionnaires in the hospitals.

In total, 1,523 questionnaires from the 8 hospitals were received (response rate = 95.1%). Out of these, a total of 1509 questionnaires were obtained (94.3%) after excluding invalid and missing data. Again, physicians aged between 24 and 60 as well as doctors under training were excluded. Here, the term physician encompassed medical practitioners from different clinical sections including surgery, obstetrics, gynecology, and pediatrics. The participants were 769 male (50.96%), 740 female (49.04%), age *M* = 36.67, and SD = 7.78. The samples were representative, with a relatively wide coverage based on characteristics.

### 2.2. Job Performance

The job performance of the physicians was evaluated using the Job Performance Scale developed in 2009 to assess job performance levels by Motowidle et al. and revised by Chinese scholars [35]. The scale comprised three subscales (task performance, interpersonal facilitation, and work engagement). The scale included 33 items, with each item, scored on a 5-point scale (1, never and 5, always). A high score indicated a higher level of job performance. In this study, the CFA of the scale indicated a good fit: *χ*^2^/df = 1.63, CFI = 0.98, TLI = 0.96, SRMR = 0.04, RMSEA = 0.04, and the Cronbach's alpha was 0.96.

### 2.3. Achievement Motivation

The 30-item Achievement Motivation Scale modified by Li et al. [36] was utilized to assess the level of achievement-motivation in physicians. The scale comprised two subscales (pursue success and avoid failure), and each subscale had 15 items. Each item was scored from 1 (completely nonconforming) to 4 (completely conforming). A high score indicated a high level of achievement motivation. In this study, the CFA of the scale indicated a good fit: *χ*^2^/df = 1.94, CFI = 0.93, TLI = 0.92, SRMR = 0.05, RMSEA = 0.05, and the Cronbach's alpha were 0.89 and 0.91.

### 2.4. Organizational Commitment

Organizational Commitment Questionnaire revised by Sharif et al. [37] was used to estimate organizational commitment. The scale comprised three subscales (emotional commitment, continuous commitment, and normative commitment). It contained 18 items, and each item is scored on a 5-point scale, where 1 denotes strongly disagree, and 5 implies strongly agree. A high score indicated a higher level of organizational commitment. Notably, the questionnaire was highly reliable and suitable for the Chinese population. In this study, the CFA of the scale indicated a good fit: *χ*^2^/df = 1.93, CFI = 0.95, TLI = 0.94, SRMR = 0.05, RMSEA = 0.05, and the Cronbach's alpha was 0.91.

### 2.5. Personality

Personality was measured using the Neuroticism, Extraversion, and Openness Personality Inventory (NEO-PI), which was introduced by the American psychologists, Costa and colleagues in 1987 and revised by Wang et al.[38]. It consisted of 25 items, with a five-point scale of 1~5 points used to divide into five subscales, including neuroticism, extraversion, openness, agreeableness, and conscientiousness. Each item consists of two words with opposite meanings; the subjects need to choose the most suitable description from each number table. If the attitude is moderate, mark it at 3. The items of neuroticism were reverse-scored. The scale was suitable for the Chinese population and had suitable validity and reliability. In this study, the CFA of the scale indicated a good fit: *χ*^2^/df = 1.28, CFI = 0.96, TLI = 0.95, SRMR = 0.05, RMSEA = 0.03, and the Cronbach's alpha is 0.84.

### 2.6. Statistical Analyses

#### 2.6.1. Preliminary Analyses

The demographic data (sex, age, education level, marital status, and professional qualifications) were assessed and compared using descriptive analysis, one-way analysis of variance (ANOVA), and an independent *t*-test. Moreover, Pearson correlation coefficients were used to examine the correlations between the study variables. Correlational analyses of job performance, achievement motivation, organizational commitment, neuroticism, extraversion, openness, agreeableness, and conscientiousness were performed using SPSS 22.0 for Windows. Statistical significance was defined at a two-tailed *p* value of less than 0.05 (*p* < 0.05).

#### 2.6.2. Mediation and Moderation Analyses

Further, the hypothesized moderated mediation model was tested using Process macro 2 (model 58) developed for SPSS [39]. This macro leverages a bootstrapping approach to estimate the conditional indirect effects of an independent variable (achievement motivation) through a mediator (organizational commitment) on a dependent variable (job performance) at five dimensions of a moderator (personality). The present study used 5000 bootstraps in and determined the mediating effect at the 95% confidence interval. This effect was presented in a forest graph using Stata. To better display the moderation effect, the procedure designed by Aiken et al. was adopted to assess the conditional indirect effects of one SD above the mean, at the mean, and one SD below the mean for the personality values used as the moderator variable of interest.

## 3. Results

### 3.1. Demographic Characteristics and Descriptive Statistics and Preliminary Analyses

Data on age, sex, marital status, education, and professional qualifications were obtained in this study. Age was classified as up to 30 years old, 31-40 years of age, and over 41 years old. Marital status was categorized as single, married, or divorced/widowed. The level of education was classified as junior college or lower, college, and postgraduate or above. Professional qualifications were categorized as primary title, intermediate title, senior vice title, senior title, and none. The effective responses in the present study were obtained from 1509 physicians (770 males [51.0%] and 739 females [49.0%]).

The characteristics of the participants and the distribution of job performance are shown in Table 1. We collected data from 770 male (51.0%) and 739 female physicians (49.0%). The average age of the study population was 36.70 ± 7.79 years (mean ± SD). The average score for job performance of the participants in this study was 126.84 ± 18.21. There were no significant differences in job performance associated with sex. Mean comparison showed there were significant associations between job performance and age (*F* = 10.930, *p* = 0.000), education (*F* = 4.574, *p* = 0.010), professional qualifications (*F* = 7.723, *p* = 0.000), and marital status (*F* = 15.495, *p* = 0.010). There is a significant difference of job performance among physicians in varied aged groups, different educational background, marital status, and the professional levels.

The means, standard deviations, and intercorrelations of all variables are presented in Table 2. Achievement motivation was positively correlated with organizational commitment (*r* = 0.43, *p* < 0.01) and job performance (*r* = 0.41, *p* < 0.01), and organizational commitment was positively correlated with job performance (*r* = 0.48, *p* < 0.01). In addition, intercorrelations among all study variables are presented separately for neuroticism, extraversion, openness, agreeableness, and conscientiousness in Table 2.

### 3.2. Moderated Mediational Analyses

**Figure 1:**
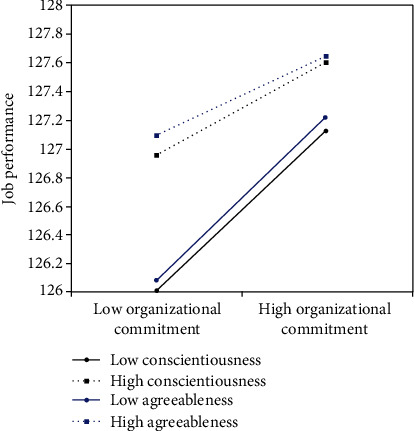
Moderating effect. Agreeableness dimension and conscientiousness dimension moderate the relationship between organizational commitment and job performance among Chinese physicians.

**Figure 2:**
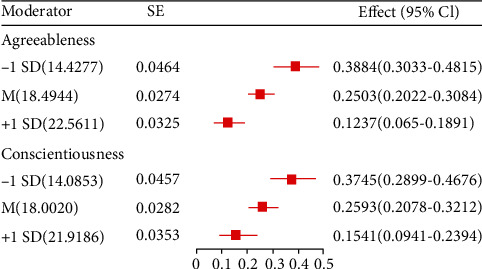
Conditional indirect effect at specific levels of the moderator when treating organizational commitment as a mediator (*N* = 1509).

Table 3 and Figure 1 presented the main results generated by Hayes' (2013) SPSS macro PROCESS, and they included mediator and dependent variable model and conditional indirect effect analysis. As can be seen from the mediator and dependent variable model, achievement motivation positively predicted organizational commitment (*β* = 0.45, *p* < 0.001), organizational commitment positively predicted job performance (*β* = 0.64, *p* < 0.001), and achievement motivation positively predicted job performance (*β* = 0.43, *p* < 0.001). These results indicated organizational commitment partially mediated the relationship between achievement motivation and job performance. Therefore, H1 and H2 were supported. Besides, the interaction of organizational commitment and personality (agreeableness and conscientiousness) showed significant effects on job performance (*β* = −0.07, *p* < 0.001; *β* = −0.06, *p* < 0.001). These results indicated that the mediating effect of organizational commitment in the association between achievement motivation and job performance was moderated by personality (agreeableness and conscientiousness). To clarify the direction and trend of moderation, the agreeableness and conscientiousness dimensions were plotted in the personality moderated effect diagram based on the Aiken et al. recommended method.  Figure 2 presents the predicted job performance value as a function of organizational commitment and personality (agreeableness and conscientiousness). Simple slope tests revealed a significant regression slope of organizational commitment on job performance in the level of agreeableness and conscientiousness dimensions being above that of 1 SD deviations (*B*_simple_ = 0.269, *p* < 0.001; *B*_simple_ = 0.319, *p* < 0.001). However, the effect of organizational commitment on job performance was much stronger in the level of agreeableness and conscientiousness dimensions being below 1 SD deviations (*B*_simple_ = 0.606, *p* < 0.001; *B*_simple_ = 0.612, *p* < 0.001). Thus, H1, H2, and H3 were supported.

**Figure 3:**
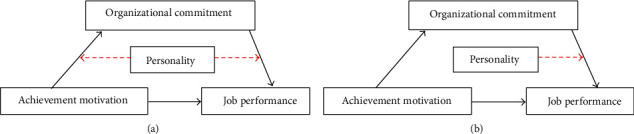
(a) The moderated mediation model applied in this study. (b) The final moderated mediation model. The link associating the achievement motivation and organizational commitment with job performance is moderated by personality traits.

This work used the bootstrap method to evaluate the conditional indirect effects of the achievement motivation level on job performance through organizational commitment, as a function of different ranges of the agreeableness and conscientiousness dimensions. The indirect effects at three levels of the agreeableness and conscientiousness dimensions were examined at 95% CIs of the bootstrap method. The conditional indirect impact on job performance originated from achievement motivation through organizational commitment (Figure 1). This effect changed according to the range of agreeableness and conscientiousness dimension which was weakest at 1 SD above the mean of the agreeableness and conscientiousness dimensions. These findings indicate that the more the points on agreeableness and conscientiousness dimensions a physician exhibits, the lower the organizational commitment mediating the effect of achievement motivation on job performance. The final moderated mediation model is presented in Figure 3(b).

## 4. Discussion

Herein, we investigated the job performance among Chinese physicians and explored a model of the relationship between achievement motivation, personality traits, organizational commitment, and job performance through a moderated mediation analysis. It was found that achievement motivation directly influences job performance of Chinese physicians, while organizational commitment partially mediates the direct effects of achievement motivation on job performance. It was further established that only agreeableness and conscientiousness moderate the strength of the relationships between achievement motivation and job performance. These findings coincide with the need for more cooperation in medical activities. Previous studies were limited to the direct effect of achievement motivation in job performance; however, these studies neglected the mechanism of this effect. Therefore, the present study reveals the mechanism of achievement motivation and job performance, geared towards providing better suggestions for hospital managers to improve the performance of doctors thereby achieving the social benefits of the hospital.

Consistent with our hypothesis, the current study found that organizational commitment was an important factor that mediated the association between achievement motivation and job performance. Specifically, achievement motivation and job performance use had a positive predicting effect on organizational commitment, which in turn predicted higher job performance. This finding is also consistent with earlier studies indicating organizational commitment as a psychosocial mechanism that may link the relationship between motivation and job performance [40, 41]. Also, this finding is not also consistent with Widarto and Anindita's study [42] where they found that there was no mediate effect in the relationship. These findings reveal a strong positive correlation between achievement motivation and job performance in physicians, which corroborates with the results from previous studies [43]. Based on the achievement need theory coined by McClelland, the achievement motivation is the desire to do well relative to the standard of excellence [44]. Employees having high achievement motivation tend to be more realistic in their career aspirations and consequently work harder and better compared to their counterparts with low achievement motivation [45]. Physicians represent a high-knowledge group, with advanced professional training, a clear career goal, high expectations in life, and value and pay more attention to individual development [46]. In our study, we established that physicians exhibiting high achievement motivation levels are motivated to succeed and more efficient at work than physicians with a low level of achievement motivation. As a result, they have a high job performance.

Notably, physicians undergo prolonged training and continuously acquire professional knowledge and clinical practice skills. They have a clear career goal and a higher level of achievement motivation [47]. Physicians with high achievement motivation are highly recognized and willing to devote extra time and energy working for their organizations with enthusiasm, hence a high organizational commitment [48]. Also, such people have high levels of emotional input to their organizations. In such an organizational environment, besides devoting themselves to work under the organization, employees take responsibilities [49]. In addition, they actively help others fronting the interests of their organizations, hence improving the performance of employees [50]. At the same time, the high organizational commitment of physicians is reflected in the importance of patient safety [51]. This spiritual concept from the internal organization guides physicians in paying more attention to patient safety. Patient safety is among the crucial indices of evaluating the performance of a physician [52].

Notably, our integrated moderated mediation analyses demonstrated general support for hypothesis 3. The results indicate that agreeableness and conscientiousness moderate the mediated effect of achievement motivation for job performance. The moderate point is in the second half of the mediation chain, i.e., the personality of the physicians affects the relationship between organizational commitment and job performance. However, neuroticism, extraversion, and openness showed no significant effect on this relationship. This is because agreeableness and conscientiousness focus more on teamwork, supporting the idea that medical activities are accomplished jointly by many departments [53]. Conversely, openness, extraversion, and neuroticism are crucial in personal performance and more prominent in the sales industry [54]. Figures 1 and 2 show the relationship between agreeableness and conscientiousness with organizational commitment and job performance. Agreeableness and conscientiousness enhance the relationship between organizational commitment and job performance. Also, the moderator role of conscientiousness personality was reported by previous studies [55, 56]. Physicians with high conscientiousness are more self-disciplined, planned, and organized. They devote themselves to every patient and have a comprehensive treatment plan; hence, they perform optimally, thereby enhancing the strength of organizational commitment and job performance [57]. Unlike in previous studies [58, 59], we found that agreeableness personality moderates job performance. This could be attributed to the study population consisting of sales employees, who draw inspiration from high competition to achieve high job performance. In contrast, the complexity and uncertainty of diseases demand physicians to consult and cooperate on multiple disciplinary teams. Physicians with high agreeableness scores are more willing to help others, pay more attention to cooperation, and prioritize organizational interests [60]. Therefore, such physicians show high job performance, translating to high numbers of improving patients. Several lines of evidence show that the management ability of medical workers promotes work performance, and people with high levels of conscientiousness and agreeableness exhibit high management ability [61]. These findings are consistent with our results.

Therefore, based on our findings, we provide recommendations for the hospital managers to improve the performance of physicians. First, we propose that hospital managers should pay close attention to the growth of young doctors. In addition to providing them with more opportunities to practice independent medical activities, more consideration should focus on their guardianship in the growth process, vigorous advocacy, and excellent practice by experienced doctors training young apprentices. Secondly, managers should utilize advanced management strategies to achieve scientific management, such as creating a positive working environment and training for career planning and education, and to improve the level of organizational commitment of physicians. Finally, managers should identify conscientious and agreeable individuals and offer them leadership roles, so that they improve organizational performance. At the same time, in the undergraduate and subsequent training of physicians, managers should strengthen the conscientiousness and agreeableness of physicians.

This study had worth mentioning limitations. First, we used a cross-sectional study design, which cannot infer the causal relationships between variables. In future studies, the use of the traced design in verifying our findings is recommended. Secondly, given the large sample size of this study, the variable measurement adopted the self-reporting method, which could result in a method effect. Therefore, we recommend a third-party evaluation of work performance and other more objective methods to collect data. Additionally, only the top 8 hospitals in Harbin were sampled. This influences the reliability of our results, and generalizing these findings in other parts of China requires validation. Therefore, future research should include other hospitals at different levels in China.

## 5. Conclusion

In conclusion, this study, for the first time, used the moderated mediation model to reveal the association between achievement motivation and job performance among Chinese physicians. It enriches the connotation of job performance-related theories and emphasizes the impacts of individual psychological factors on job performance. Also, it enriched the research scope of achievement motivation and job performance theory. We reveal that job performance and sex of physicians are not strongly correlated. Also, organizational commitment partially mediates the association between achievement motivation and job performance. Agreeableness and conscientiousness traits moderate the strength of the relationship between motivation achievement and job performance. Moreover, the mediated relationship is more robust for highly agreeable and conscious physicians compared to those with low scores of these traits.

## Figures and Tables

**Table 1:** Participants'characteristics and the distribution of job performance.

Variables	Group	*N* (%)	Job performance (*M* ± SD)	*F*/*t*	*p*
Sex					
	Male	770 (51.0)	126.16 ± 19.04	-1.499	0.134
	Female	739 (49.0)	127.56 ± 17.28		
Age					
	≤30	439 (29.1)	124.14 ± 17.76	10.930	0.000
	31-40	685 (45.4)	126.78 ± 17.57		
	≥41	385 (25.5)	130.04 ± 19.33		
Marital status					
	Single	394 (26.1)	123.84 ± 18.16	15.495	0.000
	Married/cohabiting	1051 (69.6)	128.44 ± 17.83		
	Divorced/separated/widowed	64 (4.3)	119.09 ± 20.36		
Education					
	Junior college or lower	29 (1.9)	117.04 ± 17.52	4.574	0.010
	College	328 (21.7)	127.68 ± 18.90		
	Postgraduate or above	1152 (76.4)	126.86 ± 17.97		
Professional qualifications					
	Primary title	264 (17.5)	124.80 ± 17.65	7.723	0.000
	Intermediate title	493 (32.7)	127.16 ± 18.32		
	Senior vice title	378 (25.1)	126.97 ± 17.81		
	Senior title	216 (14.3)	132.38 ± 18.09		
	None	158 (10.4)	121.42 ± 17.93		

**Table 2:** Means, standard deviations, and correlations for all study variables (*N* = 1509).

Variable	*M*	SD	1	2	3	4	5	6	7	8	9
1. Age	36.67	7.78	1								
2. Achievement motivation	3.03	10.270	.019	1							
3. Organizational commitment	63.32	10.751	.061^∗^	.429^∗∗^	1						
4. Neuroticism	15.23	3.251	-.046	.200^∗∗^	.706^∗∗^	1					
5. Extraversion	16.40	3.247	-.032	.179^∗∗^	.135^∗∗^	-.255^∗∗^	1				
6. Openness	18.66	3.580	-.002	.443^∗∗^	.597^∗∗^	.117^∗∗^	.161^∗∗^	1			
7. Agreeableness	18.49	4.062	-.043	.225^∗∗^	.109^∗∗^	.019	.637^∗∗^	.257^∗∗^	1		
8. Conscientiousness	18.00	3.918	-.015	.233^∗∗^	.110^∗∗^	.003	.574^∗∗^	.232^∗∗^	.808^∗∗^	1	
9. Job performance	126.72	18.160	.138^∗∗^	.410^∗∗^	.483^∗∗^	.187^∗∗^	.232^∗∗^	.574^∗∗^	.388^∗∗^	.384^∗∗^	1

**Table 3:** Conditional process analysis.

	*β*	SE	*t*	*p*
Mediator variable model for predicting				
Organizational commitment				
Constant	61.98	0.26	237.93	0.000
Achievement motivation	0.45	0.02	18.21	0.000
Dependent variable model for predicting				
Job performance				
Constant	84.70	2.59	32.73	0.000
Achievement motivation	0.43	0.04	9.90	0.000
Organizational commitment	0.64	0.41	15.63	0.000
Agreeableness	0.36	0.04	9.03	0.000
Organizational commitment×agreeableness	-0.07	0.01	-7.91	0.000
Conscientiousness	0.35	0.04	8.70	0.000
Organizational commitment×conscientiousness	-0.06	0.01	-6.38	0.000

## Data Availability

The datasets and/or analyses from the current study are available from the corresponding authors upon reasonable request.

## Abbreviations

CFA: Confirmatory factor analysis.
